# Supplementary material for: A Critical View of the Application of the APEX Software (Aqueous Photochemistry of Environmentally-Occurring Xenobiotics) to Predict Photoreaction Kinetics in Surface Freshwaters
Source: Molecules. 2019 Dec 18;25(1):9. doi: 10.3390/molecules25010009 (PMC7017383; doi:10.3390/molecules25010009)
Supplement: Supplementary file 1 [file molecules-25-00009-s001.zip › 3.2.4_gcc-4.4.0.zip!/3.2.4_gcc-4.4.0/3.2.4_gcc-4.4.0/doc/octave/html/interpreter/Quiver-group.html]

Quiver group - Untitled


Next: Scatter group,
Previous: Line series,
Up: Object Groups


---

##### 15.2.8.7 Quiver group

Quiver series objects are created by the `quiver` or `quiver3`
functions. Each `hggroup` element of the series contains three line
objects as children representing the body and head of the arrow,
together with a marker as the point of original of the arrows. The
properties of the quiver series are

`autoscale` `autoscalefactor`: Flag whether the length of the arrows is scaled or defined directly from the u, v and w data. If the arrow length is flagged as being scaled by the `autoscale` property, then the length of the autoscaled arrow is controlled by the `autoscalefactor`. `maxheadsize`: This property controls the size of the head of the arrows in the quiver series. The default value is 0.2. `showarrowhead`: Flag whether the arrow heads are displayed in the quiver plot. `color`: The RGB color or color name of the line objects of the quiver. See Colors. `linewidth` `linestyle`: The line width and style of the line objects of the quiver. See Line Styles. `marker` `markerfacecolor` `markersize`: The line and fill color of the marker objects at the original of the arrows. See Colors. `xdata` `ydata` `zdata`: The origins of the values of the vector field. `udata` `vdata` `wdata`: The values of the vector field to plot. `xdatasource` `ydatasource` `zdatasource` `udatasource` `vdatasource` `wdatasource`: Data source variables.
